# Identification of anoikis-related genes to develop a risk model and predict the prognosis and tumor microenvironment in rectal adenocarcinoma

**DOI:** 10.3389/fgene.2025.1604541

**Published:** 2025-08-18

**Authors:** Bing Zhao, Xuegui Tang

**Affiliations:** ^1^ Department of Integrated Traditional and Western Medicine Anorectal, Affiliated Hospital of North Sichuan Medical College, Nanchong, China; ^2^ Anorectal Department, Affiliated Hospital of Chengdu University of Traditional Chinese Medicine, Chengdu, China

**Keywords:** anoikis-related genes, risk model, rectal adenocarcinoma (READ), prognosis, tumor microenvironment

## Abstract

**Background:**

Rectal adenocarcinoma (READ) is a common malignant tumor. This study aims to establish a risk model based on anoikis-related genes (ARGs) to predict prognosis and the tumor microenvironment in READ.

**Methods:**

Transcriptomic data and clinical data downloaded from the TCGA and GEO databases were used for differential analysis and Cox regression analysis. An ARGs-based prognostic risk model was constructed for READ. The survival curves and ROC curves were plotted to determine the predictive ability of the model for READ patients. The model was externally validated in the GSE87211 dataset. A nomogram, immune analysis, drug sensitivity analysis, and functional enrichment analysis were also performed to comprehensively validate the model.

**Results:**

The risk model included 6 prognostic genes (ALDH1A1, BRCA1, GSN, KRT17, SCD, and SNCG). Kaplan-Meier curves for the TCGA training cohort (P < 0.0001), testing cohort (P = 0.018), and GSE87211 dataset (P = 0.036) showed better prognoses in the low-risk group. The AUC for 1-year, 3-year, and 5-year overall survival in the TCGA training cohort, testing cohort, and GSE87211 dataset were (0.962, 0.923, 0.956), (0.887, 0.838, 0.833), and (0.73, 0.817, 0.743), respectively. The nomogram showed that the risk score served as an independent predictor of overall survival. Drug sensitivity analysis revealed differences in the IC50 values of OSI-027, PLX-4720, UMI-77, and Sapitinib between the high-risk and low-risk groups. Immune microenvironment analysis suggested distinct differences in immune cells between the two risk groups. Enrichment analysis revealed that these prognostic ARGs were primarily enriched in pathways and biological processes related to tumorigenesis.

**Conclusion:**

The risk model of ARGs can effectively predict READ prognosis and provide potential therapeutic targets.

## 1 Introduction

Rectal cancer is a prevalent malignancy of the digestive tract and the third prevalent cancer globally, as the latest epidemiological survey states (Liu et al., 2023). Rectal adenocarcinoma (READ) is the most prevalent type of rectal cancer, located between the dentate line and the junction of the rectum and sigmoid colon. The absence of obvious symptoms in the early stages makes the diagnosis usually established in the middle or late stages, which seriously influences treatment outcomes. Despite advances in surgical techniques and improvements in radiotherapy and chemotherapy, the 5-year overall survival (OS) for READ remains unfavorable (Cui et al., 2021). Recent studies have demonstrated that PLAGL2, ZNF337, and ALG10 are involved in the pathogenesis of READ and are associated with chemotherapy sensitivity (Zhao P. et al., 2023). In addition, heterogeneous nuclear ribonucleoproteins (hnRNPs) are all upregulated in READ tissues, and immunohistochemistry has confirmed the overexpressed levels of hnRNPA1, hnRNPA2B1, hnRNPK, hnRNPC, hnRNPR, and hnRNPU in READ (Li et al., 2022). While studies have identified some genes and proteins involved in READ development and potential as therapeutic targets, the specific biological markers or molecular mechanisms underlying the pathogenesis of READ are not yet fully clarified. Therefore, exploring genetic targets and molecular pathways of READ is crucial for its prevention, prognostic evaluation, and treatment.

Apoptosis is a genetically controlled, orderly process of cell death that occurs autonomously and maintains internal homeostasis by preventing cell functions. Anoikis is a form of apoptosis, activated by the removal of cells, and is a regulated cell death mechanism involving intrinsic and extrinsic pathways (Adeshakin et al., 2021). Anoikis is crucial in tissue homeostasis, disease progression, and tumor metastasis. Anoikis-related genes (ARGs) are pivotal in tumorigenesis, progression, and prognosis. A previous study has established a prognostic risk model for hepatocellular carcinoma (HCC) based on five ARGs (BAK1, SPP1, BSG, PBK, and DAP3). These genes are highly associated with tumorigenesis and immune cell infiltration and show strong predictive performance for OS in HCC patients (Chen et al., 2023). Another study has developed a risk model based on 16 ARGs and found that these genes can regulate diverse biological processes of lung adenocarcinoma and are effective in predicting patient prognosis (Diao et al., 2023). In addition, ARGs trigger the occurrence and progression of prostate cancer and can predict the risk of postoperative recurrence (Zhao X. et al., 2023). Although many studies have unveiled the involvement of ARGs in cancer development, their molecular mechanisms in the pathogenesis of READ remain elusive.

In this study, transcriptomic data and clinical data downloaded from TCGA and GEO databases were adopted for Cox regression analysis. A prognostic risk model based on key ARGs was constructed for READ, and a nomogram was developed, followed by enrichment analysis, immune microenvironment analysis, and drug sensitivity analysis. This promising prognostic risk model for READ may improve early diagnosis, prognostic evaluation, and treatment for READ patients.

## 2 Materials and methods

### 2.1 Data download and preprocessing

Transcriptomic data and clinical data for READ patients were downloaded from the UCSC Xena database (https://xenabrowser.net/datapages/), including 166 tumor tissue samples and 10 normal tissue samples. ARG data were downloaded from GeneCards (https://www.genecards.org/), including 919 genes. Differential expression analysis was conducted on the genes downloaded from TCGA, with a cutoff of an absolute logFC >1 and FDR <0.05. The distribution of upregulated and downregulated genes was visualized in volcano plots with the “ggplot2” package, and then differentially expressed genes (DEGs) were selected. Key coding genes were selected from 919 ARGs, and the intersecting genes between DEGs and key ARGs were determined using the “VennDiagram” package, with a Venn diagram generated. Survival data for READ patients were downloaded from UCSC Xena (https://xenabrowser.net/datapages/), including 178 samples. The survival data were merged with the gene expression matrix, resulting in 157 tumor samples with key differentially expressed ARGs (DE-ARGs).

### 2.2 Establishment and evaluation of the prognostic model

The dataset of differentially expressed samples was randomized into a training cohort and a testing cohort at a 2:1 ratio. Univariate Cox regression analysis was performed on the training cohort using the “survival” package, and genes with P < 0.05 were selected. A forest plot was generated using the “forestplot” package. LASSO regression was conducted using the “glmnet” package, and genes with coefficients shrunk to zero were selected. A 10-fold cross-validation approach was used to determine the optimal penalty parameter (λ), corresponding to the minimum partial likelihood deviance, resulting in 9 genes. Multivariate Cox analysis was then conducted to identify 6 prognostic genes and establish a prognostic risk model. The risk score was calculated using the following formula: ∑(Coef × xi), where xi represents the normalized expression of target gene i, and Coef represents the regression coefficient (Zhang et al., 2024). The training cohort and testing cohort were allocated to high-risk and low-risk groups based on the median risk score, and Kaplan-Meier (K-M) curves were plotted. ROC curves were generated using the “time ROC” function. Survival interval plots were drawn using the “ggplot” package. Gene expression similarity maps for the two cohorts were analyzed via the “ggpubr” and “reshape2” packages. Finally, complex heatmaps were plotted using the “ComplexHeatmap” package.

### 2.3 External validation of the model

Transcriptome data and clinical data for READ patients were downloaded from the GEO (https://www.ncbi.nlm.nih.gov/geo/). The GSE87211 (Platform: GPL13497) dataset, including 203 tumor samples and 160 normal samples, was used for external validation. Multivariate Cox regression analysis was performed on the dataset, and the samples were allocated into high- and low-risk groups based on the risk score. Forest plots, K-M curves, and ROC curves were generated. Survival interval distribution plots, differential gene expression similarity maps, and complex heatmaps were also plotted. To further assess the robustness of the model, the GSE133057 (Platform: GPL6102) dataset, which included 33 tumor samples, was used for external validation. K-M curves and ROC curves were subsequently generated.

### 2.4 Construction of the nomogram

The risk score was combined with clinical features (age, sex, TNM stages) of TCGA-READ patients to construct a nomogram for predicting 1- to 5-year OS. The nomogram was generated using the “nomogram” function from the “rms” package, and OS probabilities for 1, 2, 3, 4, and 5 years were evaluated. To confirm the predictive accuracy of the nomogram, a calibration curve was plotted. Univariate and multivariate Cox regression analyses were performed to examine the independent predictive accuracy of risk scores and clinical features. K-M curves were generated for each clinical feature. Risk scores under different clinical features were compared to assess the consistency across various clinical features.

### 2.5 Functional enrichment analysis

To explore the mechanisms and functions of key ARGs (Diao et al., 2023), Gene Ontology (GO) and Kyoto Encyclopedia of Genes and Genomes (KEGG) enrichment analyses were performed using the “clusterProfiler” R package. Functional pathways or processes were defined as significantly enriched when P < 0.05.

### 2.6 Immune microenvironment analysis

Gene Set Variation Analysis (GSVA) is a non-parametric and unsupervised technique that evaluates the enrichment level of transcriptomic genes. By integrating scores for the genes, GSVA transforms gene-level changes into pathway-level changes, thus analyzing the biological functions (Zhao P. et al., 2023). ssGSEA is an algorithm within the GSVA package, widely used to evaluate immune infiltration. The “ssgseaParam” function was utilized to assess differences in immune infiltration levels between the normal and disease groups. The “ssGSEA” algorithm was used to generate ssGSEA plots to evaluate expression differences among various immune cells. Heatmaps were plotted using the “msigbr” and “pheatmap” R packages to assess the expression levels of different pathways. Correlation analysis was performed using the “psych” and “ggplot2” R packages to explore the association between prognostic genes and immune cells (Liu et al., 2023). The “corrplot” R package was utilized for correlation analysis between various immune cells (Liu et al., 2022). Additionally, the “IOBR” R package was used for “ESTIMATE” analysis to unveil differences in stromal scores, immune scores, and tumor purity between the high-risk and low-risk groups. “TIDE” analysis was conducted online at TIDE (http://tide.dfci.harvard.edu/) to estimate the potential for immune evasion and the likelihood of benefiting from immunotherapy. The correlation analysis was conducted using the “psych” and “ggplot2” R packages to evaluate the relationship between prognostic genes and immune checkpoint genes. The tumor mutational burden (TMB) score between the high-risk and low-risk groups was calculated using the “maftools” package.

### 2.7 Drug sensitivity

Drug sensitivity analysis was conducted using the “oncoPredict” R package for 198 drugs to assess IC50 values of conventional or targeted drugs between the high-risk and low-risk groups. Then sensitive drugs were selected, and correlation analysis between drugs and prognostic genes was conducted.

### 2.8 Validation of prognostic genes

Human Protein Atlas (HPA) (https://www.proteinatlas.org/) online database was applied for validation to evaluate the protein levels of prognostic genes in tumors and adjacent normal tissues. Gene Expression Profiling Interactive Analysis (GEPIA) (http://gepia.cancer-pku.cn/) online database was used to quantitatively analyze the differential expression of prognostic genes in tumor patients and normal individuals. The TISCH2 (http://tisch.comp-genomics.org/home/) database was also used for validation. This resource allowed for single-cell resolution analysis of the complex components of the tumor microenvironment (TME), helping to uncover cellular heterogeneity and interactions in the TME, thus improving tumor treatment strategies.

### 2.9 Data analysis

Data analyses were performed using R 4.4.1, and P < 0.05 implied statistical significance. K-M curve was used to determine the predictive value of the model for OS. The log-rank test was adopted to show significant differences in survival curves. ROC curves were drawn to verify the predictive accuracy of the model. Pearson correlation analysis was employed to determine the link between genes and clinical data. Significance was manifested as *P < 0.05; **P < 0.01; ***P < 0.001, while ns implied no significance.

## 3 Results

### 3.1 Acquisition of DEGs

**FIGURE 1:**
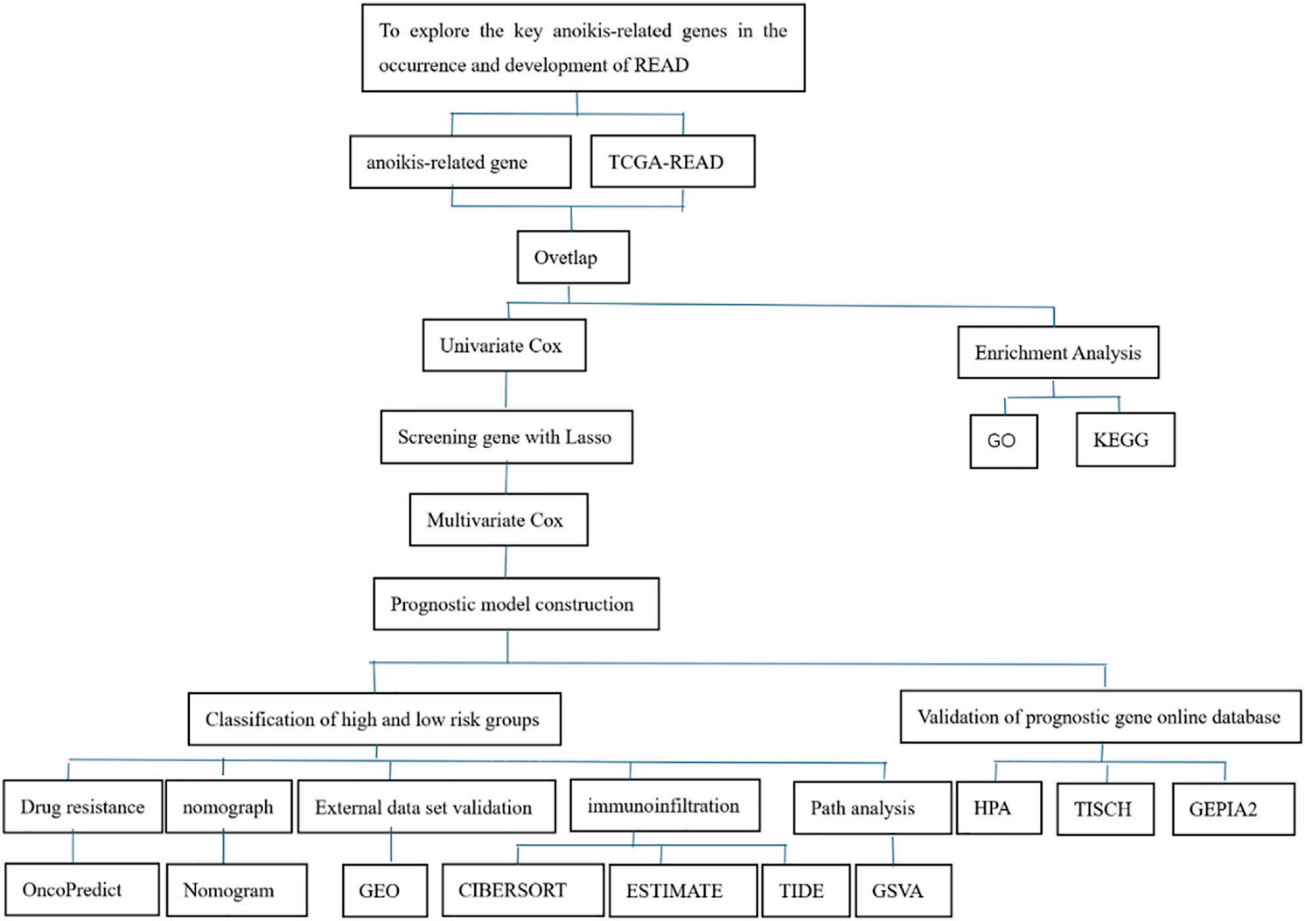
Flowchart of the study design.

**FIGURE 2:**
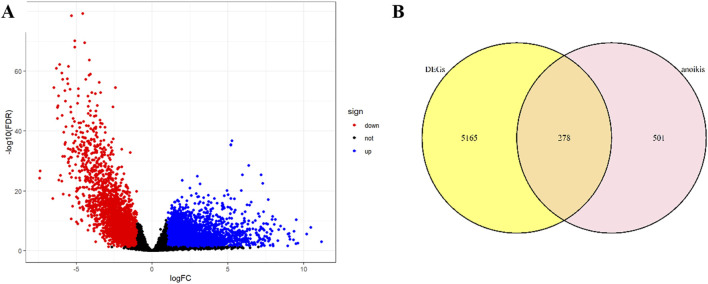
Volcano plot and Venn diagram: **(A)** Volcano plot; **(B)** Venn diagram.

The design flowchart is listed in Figure 1. A total of 5165 tumor DEGs were downloaded from TCGA and visualized in a volcano plot (Figure 2A). The red area on the left represented downregulated DEGs, while the blue area on the right represented upregulated DEGs. The intersection of DEGs and key ARGs was taken to create a Venn diagram (Figure 2B), yielding 278 key DE-ARGs.

### 3.2 Functional enrichment analysis

**FIGURE 3:**
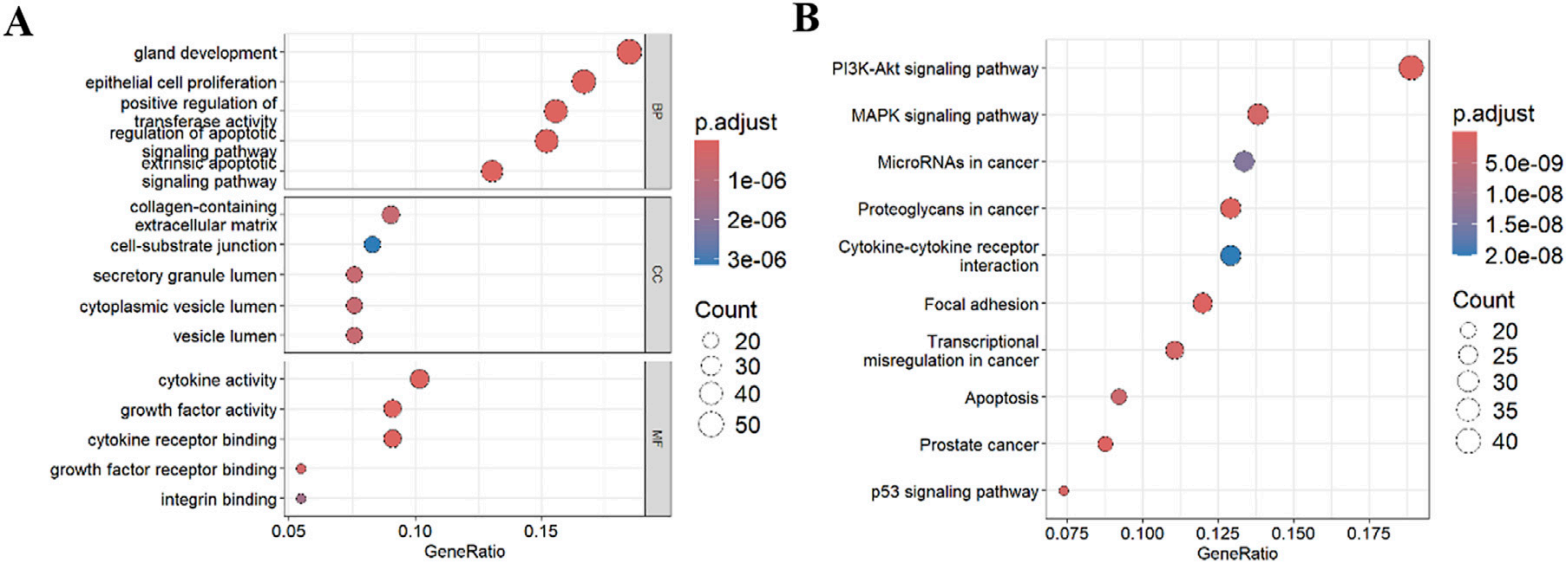
Functional enrichment analysis. **(A)** GO analysis; **(B)** KEGG analysis. BP: Biological Process; CC: Cellular Component; MF: Molecular Function.

GO enrichment analysis (Figure 3A) demonstrated that DE-ARGs were highly enriched in the following biological processes: gland development, epithelial cell proliferation, and positive regulation of transferase activity. They were also highly enriched in the following cellular components: collagen-containing extracellular matrix, cell-substrate junction, and secretory granule lumen. As for molecular functions, DE-ARGs were highly enriched in cytokine activity, growth factor activity, and cytokine receptor binding. Further KEGG analysis (Figure 3B) showed that DE-ARGs were highly enriched in the PI3-Akt pathway, MAPK pathway, and microRNAs in cancer. These results suggest that DE-ARGs are highly enriched in cancer-related pathways, indicating their involvement in tumorigenesis.

### 3.3 Evaluation of the prognostic risk model

**FIGURE 4:**
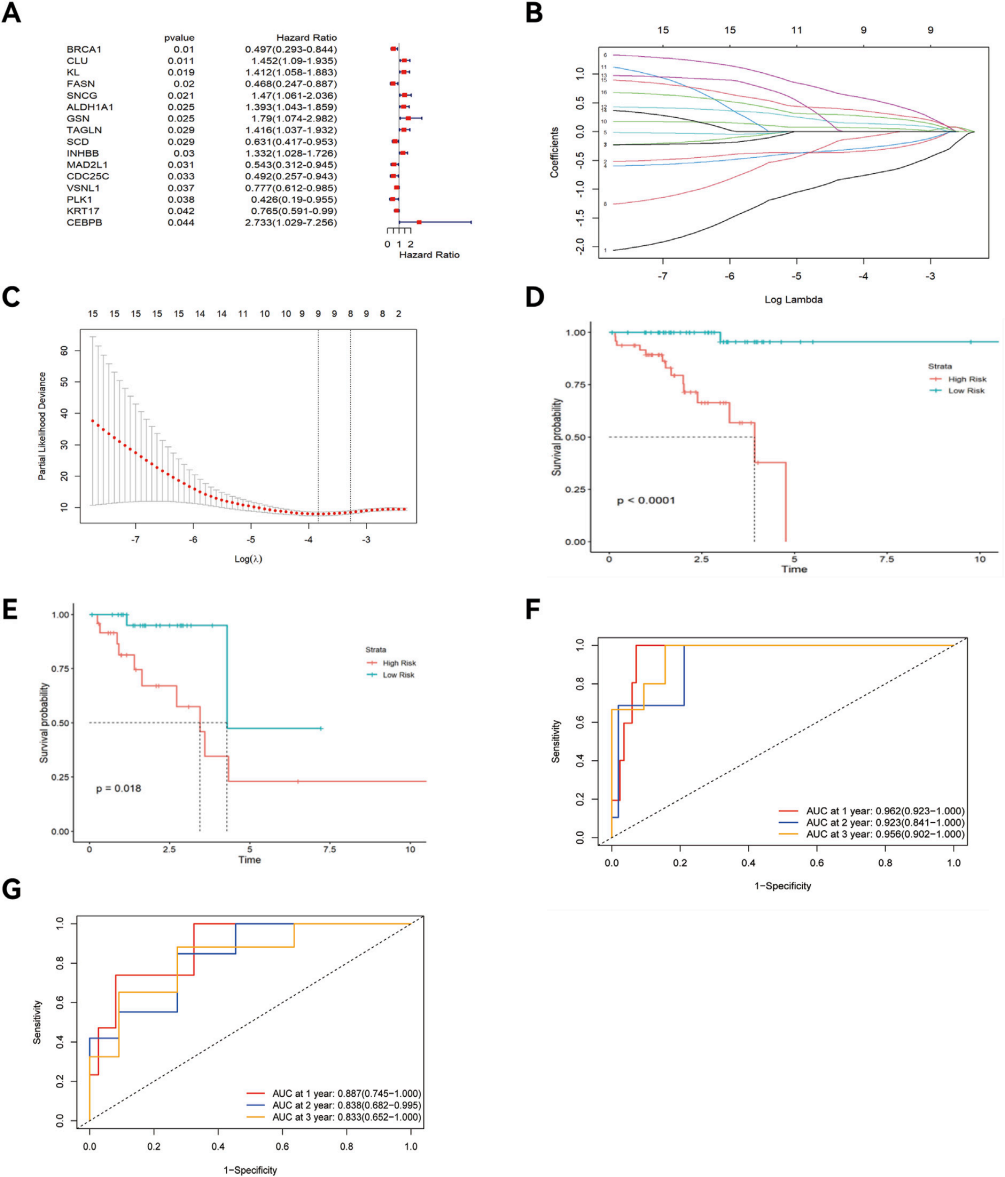
Construction of the prognostic risk model, K-M curve, and ROC curve. **(A)** Forest plot; **(B)** Lasso regression plot; **(C)** Cross-validation plot; K-M curve of the training cohort **(D)** and testing cohort **(E)**; “Time ROC” curve of the training cohort **(F)** and testing cohort **(G)**.

**FIGURE 5:**
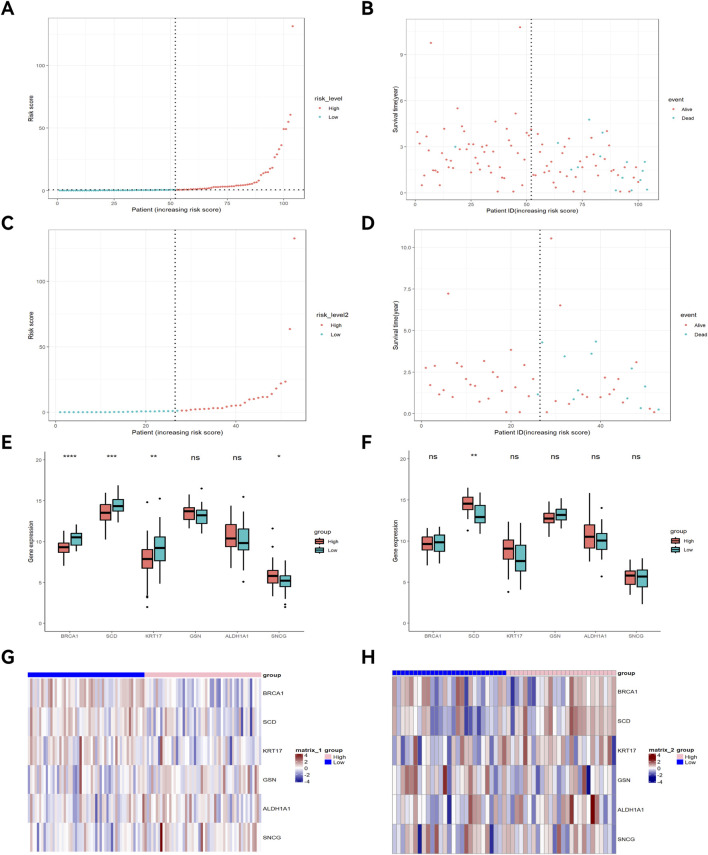
Survival interval plot, gene expression similarity plot, and heatmap. Risk score distribution plot of the training cohort **(A)** and testing cohort **(C)**; Survival interval plot of the training cohort **(B)** and testing cohort **(D)**; Gene expression similarity plot of the training cohort **(E)** and testing cohort **(F)**; **(G)** Heatmap of the training cohort; **(H)** Heatmap of the testing cohort. *p < 0.05; **p < 0.01; ***p < 0.001.

Univariate Cox regression analysis identified 16 key ARGs in the training cohort (Figure 4A). LASSO analysis (Figure 4B) and cross-validation (Figure 4C) then selected 9 key ARGs. Multivariate Cox regression and stepwise regression analyses then identified 6 prognostic genes, which were used to construct the prognostic risk model. K-M curves (Figure 4D) revealed better prognoses in the low-risk group in the training cohort (P < 0.0001). In the testing cohort, multivariate Cox regression analysis and K-M curves (Figure 4E) showed the low-risk group exhibited better prognoses (P = 0.018). The areas under the ROC curves (AUCs) for 1-year, 3-year, and 5-year OS in the training set were 0.962 (95% CI: 0.923–1.000), 0.923 (95% CI: 0.841–1.000), and 0.956 (95% CI: 0.902–1.000) (Figure 4F), and 0.887 (95% CI: 0.745–1.000), 0.838 (95% CI: 0.682–0.995), and 0.833 (95% CI: 0.652–1.000) in the testing set (Figure 4G). The risk score distribution and survival interval plots (Figures 5A–D) found that as the risk score elevated, the mortality rate of READ patients increased. Gene expression similarity plots (Figures 5E,F) and complex heatmaps (Figures 5G,H) indicated differential expression of prognostic genes between the high-risk and low-risk groups in both the training and testing cohorts.

### 3.4 External validation of the model

**FIGURE 6:**
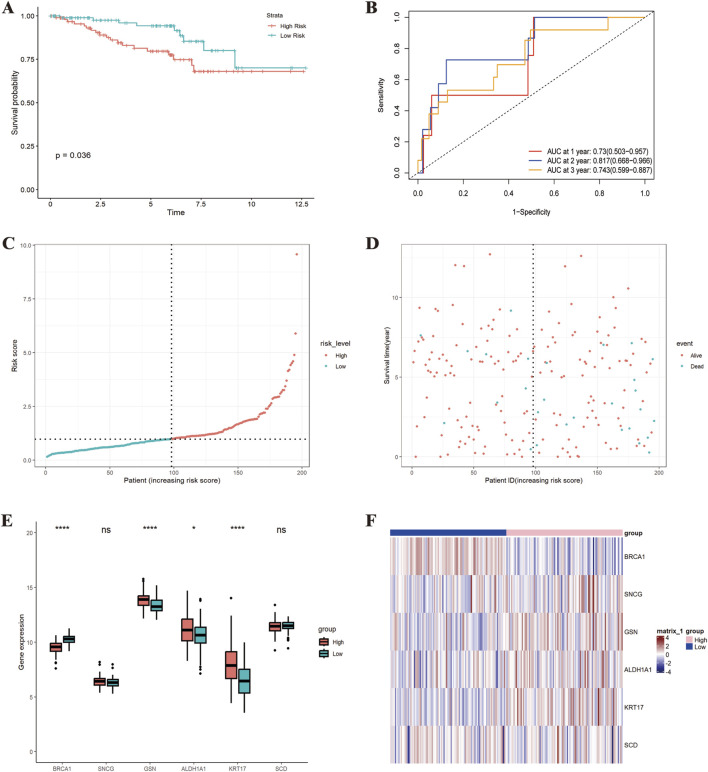
External validation of the prognostic risk model (GSE87211 dataset). **(A)** K-M curve; **(B)** “Time ROC” curve; **(C)** Risk score distribution plot; **(D)** Survival interval plot; **(E)** Gene expression similarity plot; **(F)** Heatmap. *p < 0.05; **p < 0.01; ***p < 0.001; ****p < 0.0001.

In the GSE87211 dataset, the K-M curve showed better prognoses in low-risk cohorts (P = 0.036) (Figure 6A). The AUC for 1-year, 3-year, and 5-year OS was 0.73 (95% CI: 0.503–0.957), 0.817 (95% CI: 0.668–0.966), and 0.743 (95% CI: 0.599–0.887), suggesting favorable predictive capability (Figure 6B). As risk scores increased, the mortality rate elevated (Figures 6C,D). The prognostic genes displayed differential expression between the high-risk and low-risk groups (Figures 6E,F). In the GSE133057 dataset, the K-M curve showed better prognoses in low-risk cohorts (P = 0.007) (Supplementary Figure S1A). The AUC for 1-year, 3-year, and 5-year OS was 0.968 (95% CI: 0.905–1.000), 1 (95% CI: 1.000–1.000), and 0.792 (95% CI: 0.483–1.000), suggesting favorable predictive capability (Supplementary Figure S1B)

### 3.5 Construction of the nomogram

**FIGURE 7:**
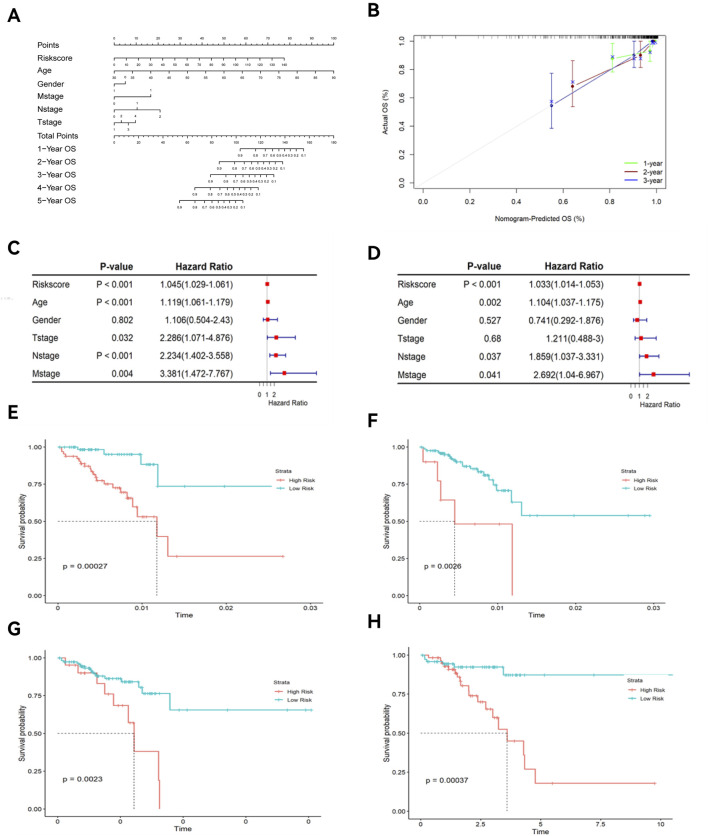
Nomogram, calibration curve, and K-M curves of clinical features. **(A)** Nomogram; **(B)** Calibration curve; **(C)** Univariate forest plot; **(D)** Multivariate forest plot; **(E)** K-M curve of age; **(F)** K-M curve of T stage; **(G)** K-M curve of M stage; **(H)** K-M curve of N stage.

**FIGURE 8:**
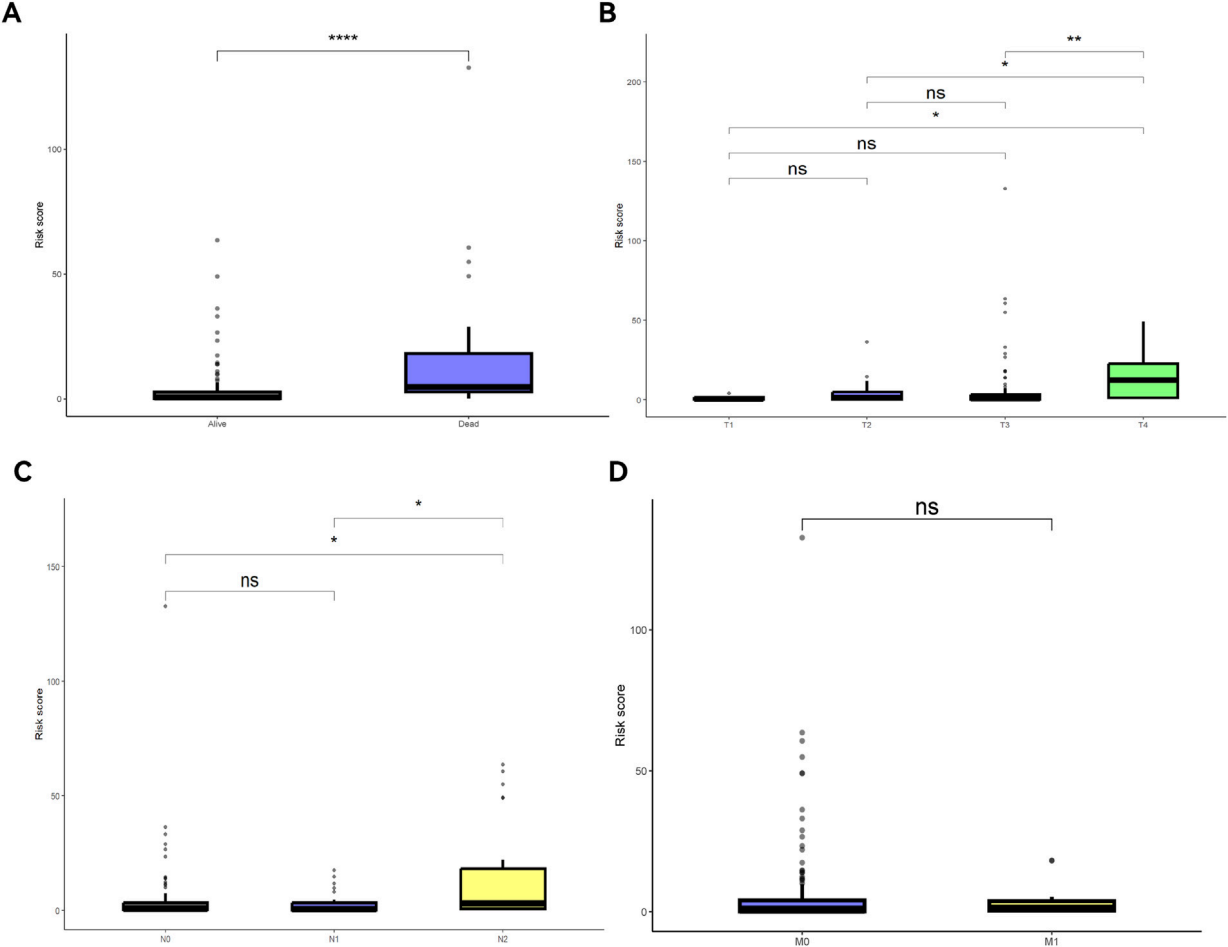
Risk scores under different clinical feature statuses. **(A)** Survival and death risk scores; **(B)** Risk scores across different T stages; **(C)** Risk scores across different N stages; **(D)** Risk scores across different M stages.

The nomogram and calibration curves (Figures 7A,B) showed that risk scores could accurately predict the 1- to 5-year OS of READ patients. Univariate and multivariate Cox regression analyses (Figures 7C,D) revealed that risk scores, age, and TNM stage could serve as independent predictors of OS (P < 0.05). Among these factors, risk scores showed a good ability to predict OS (P < 0.0001). K-M curve (Figures 7E–H) demonstrated that both age and TNM stage had predictive power for OS. The risk scores were different across patient survival statuses, T stages, and N stages (Figures 8A–D).

### 3.6 Immune microenvironment analysis

**FIGURE 9:**
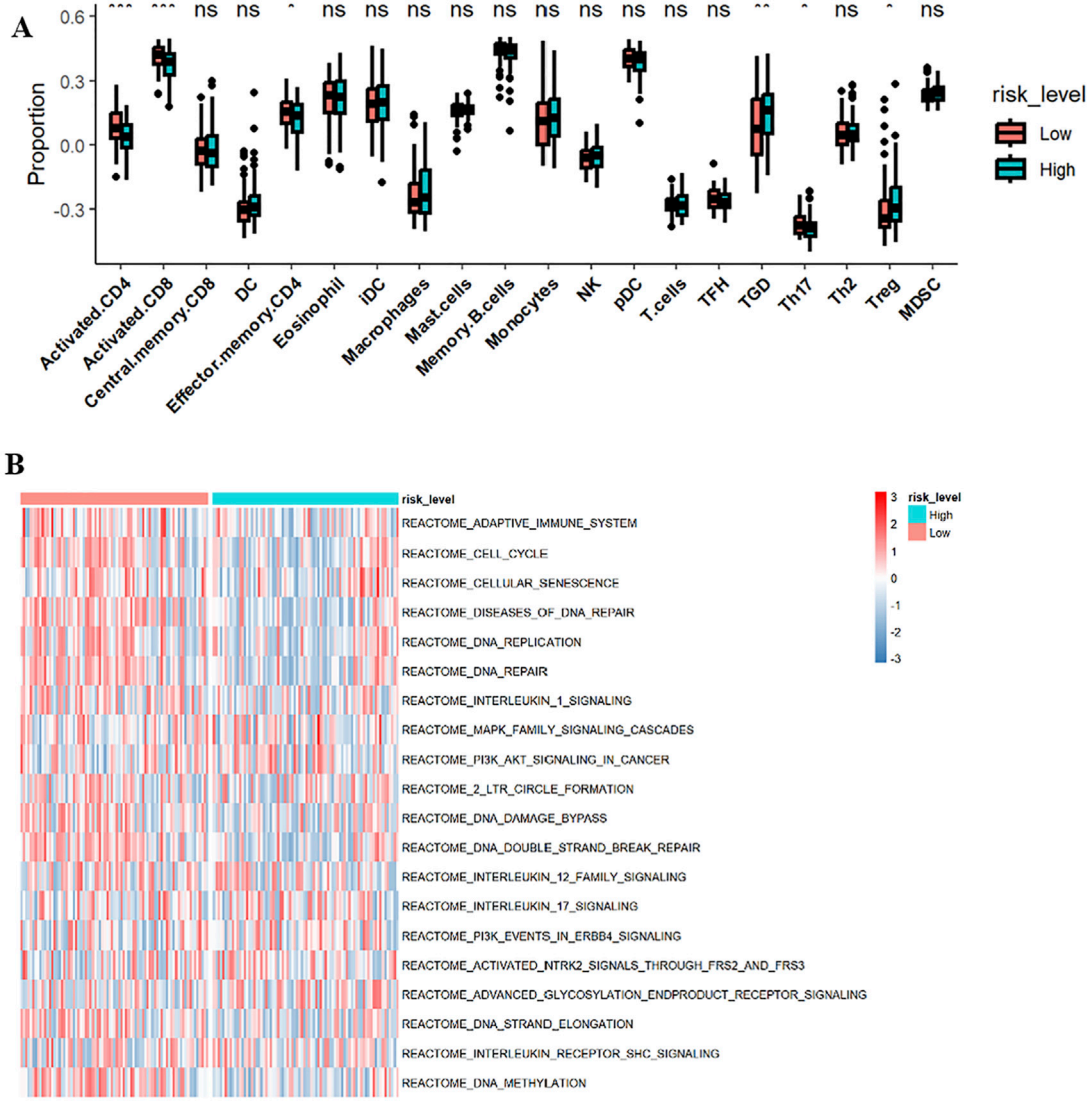
ssGSEA and Heatmap. **(A)** ssGSEA; **(B)** Heatmap. *p < 0.05; **p < 0.01; ***p < 0.001.

**FIGURE 10:**
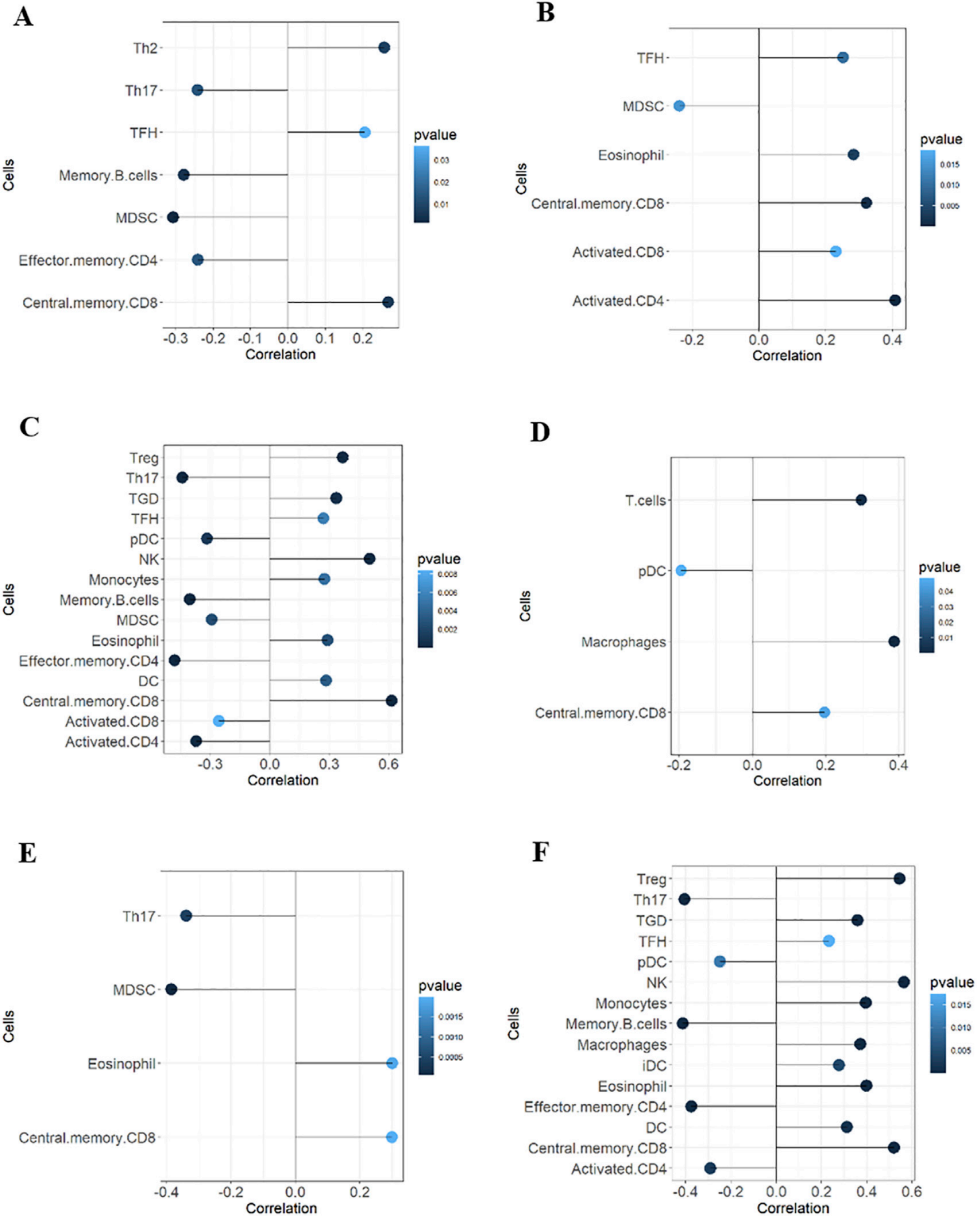
Correlation analyses between prognostic ARGs and immune cells. Correlations of ALDH1A1 **(A)**, BRCA1 **(B)**, GSN **(C)**, KRT17 **(D)**, SCD **(E)**, and SNCG **(F)** with immune cell content.

**FIGURE 11:**
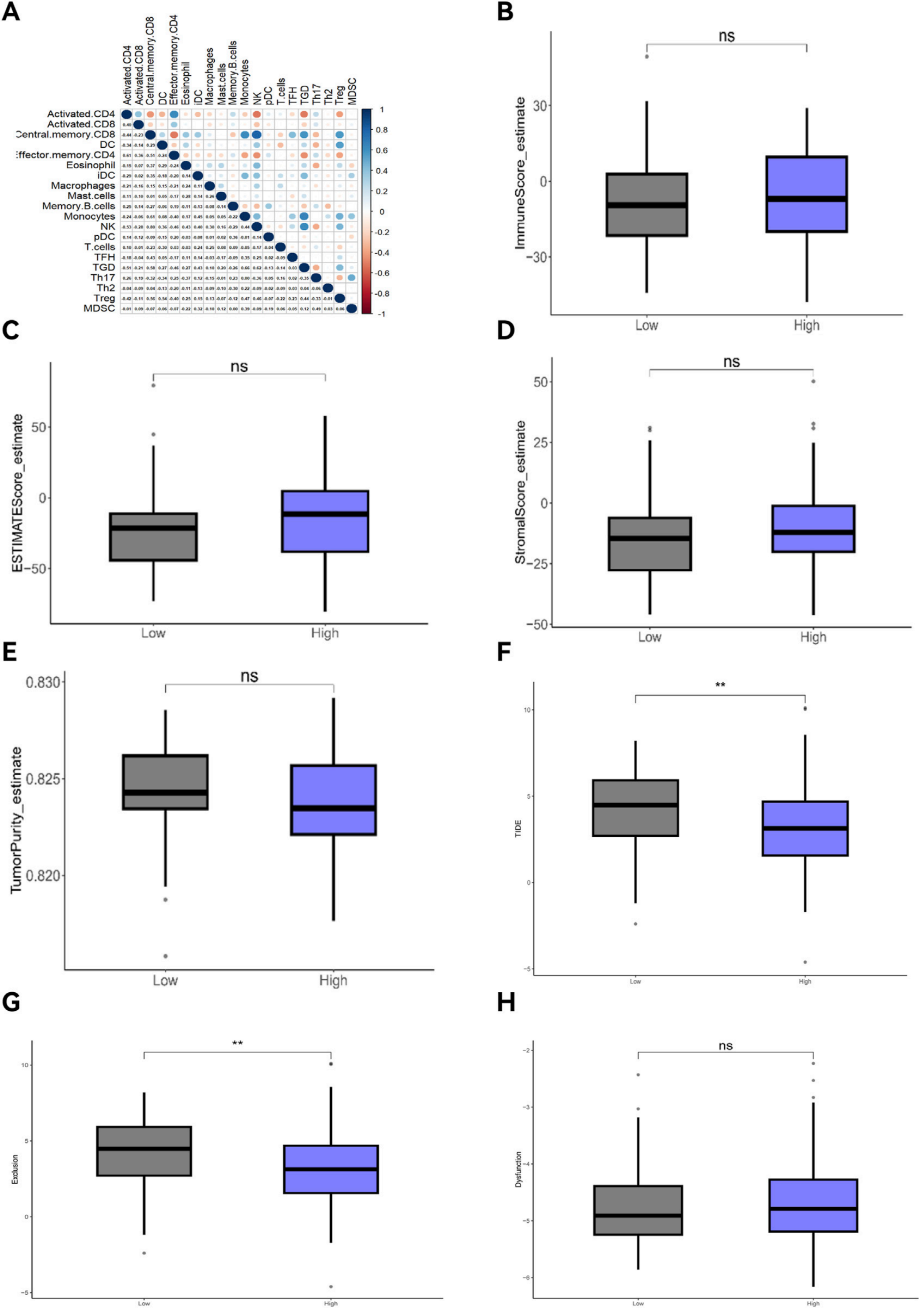
Correlation analyses of immune cells, ESTIMATE, and TIDE. **(A)** Correlation analysis of immune cells; **(B)** Tumor immune score; **(C)** ESTIMATE score; **(D)** Immune mechanism score; **(E)** Tumor purity score; **(F)** TIDE score; **(G)** Exclusion; **(H)** Dysfunction.

GSVA analysis results are shown in the ssGSEA plot (Figure 9A). “activated.CD4,” “activated.CD8,” “effector.memory.CD4,” and “Th17” were under-expressed in high-risk cohorts, while “TGD” and “Treg” were highly expressed. Among 20 tumor-related pathways, most pathways exhibited expression differences between the high-risk and low-risk groups (Figure 9B). A correlation analysis of prognostic ARGs and immune cell content (Figures 10A–F) demonstrated that ALDH1A1, BRCA1, GSN, KRT17, SCD, and SNCG were positively correlated with central.memory.CD8; ALDH1A1, BRCA1, and GSN were positively correlated with TFN and negatively correlated with MDSC; ALDH1A1, GSN, SCD, and SNCG were negatively correlated with Th17; GSN, KRT17, and SNCG were negatively correlated with PDC. Additionally, activated CD8 was positively correlated with activated CD4; T cells were positively correlated with effector.memory.CD4 and negatively correlated with PDC; Th2 was negatively correlated with PDC and positively correlated with TFH (Figure 11A). Furthermore, “ESTIMATE” analysis (Figures 11B–E) indicated no significant differences between the high-risk and low-risk groups. The “TIDE” analysis results (Figures 11F–H) revealed higher TIDE scores in the low-risk group. The correlation analysis between prognostic genes and immune checkpoint genes revealed that ALDH1A1, BRCA1, GSN, SCD, and SNCG were significantly associated with the immune checkpoint genes CD274 and CTLA-4 (Supplementary Figures S2A–J), whereas KRT17 showed no correlation with CD274 and CTLA-4 (Supplementary Figures S2K–L). There was no significant difference in the TMB score between the high-risk and low-risk groups (Supplementary Figure S3).

### 3.7 Drug sensitivity

**FIGURE 12:**
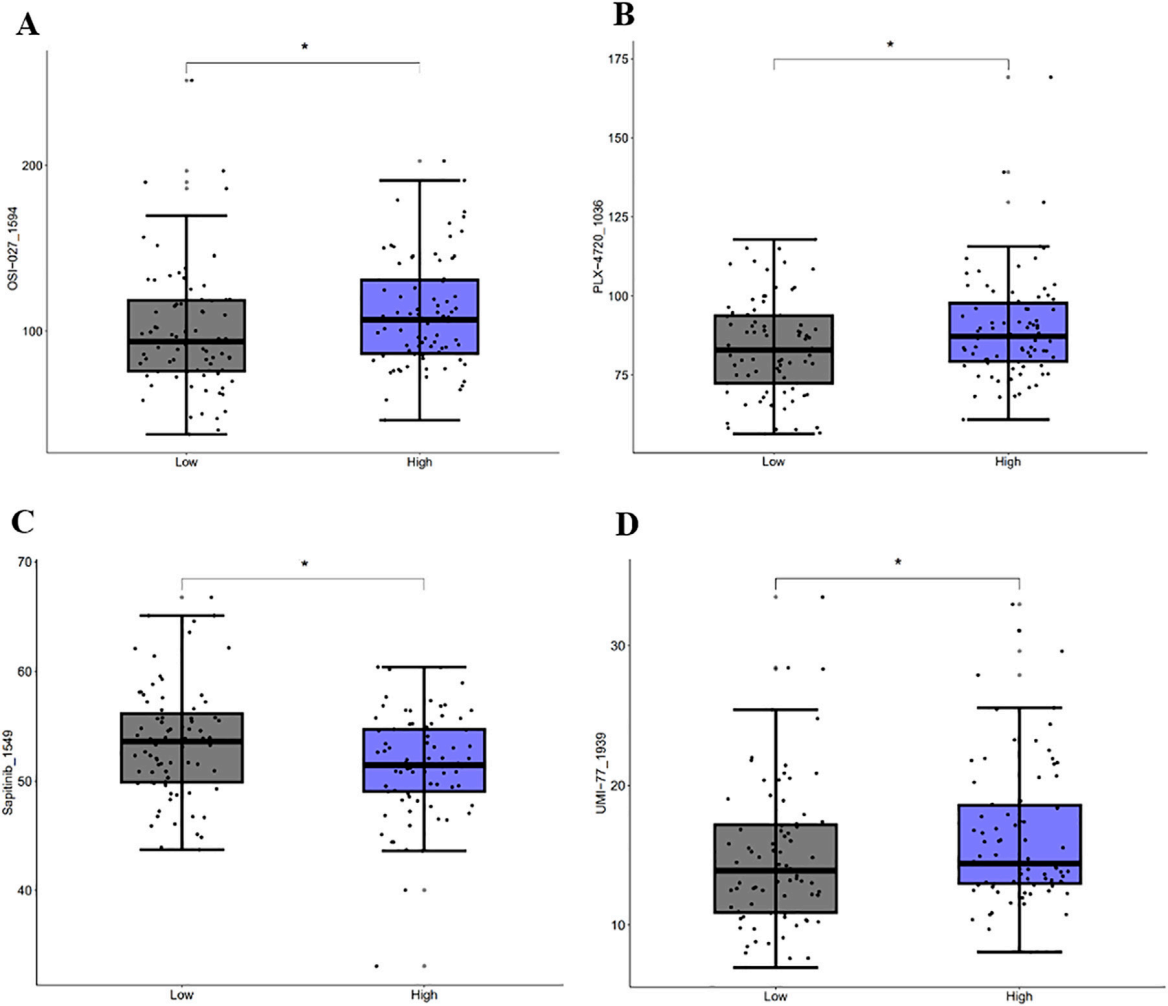
Drug sensitivity analysis. Sensitivity analysis of OSI-027_1594 **(A)**, PLX-4720_1036 **(B)**, Sapitinib_1549 **(C)**, and UMI-77_1939 **(D)**.

**FIGURE 13:**
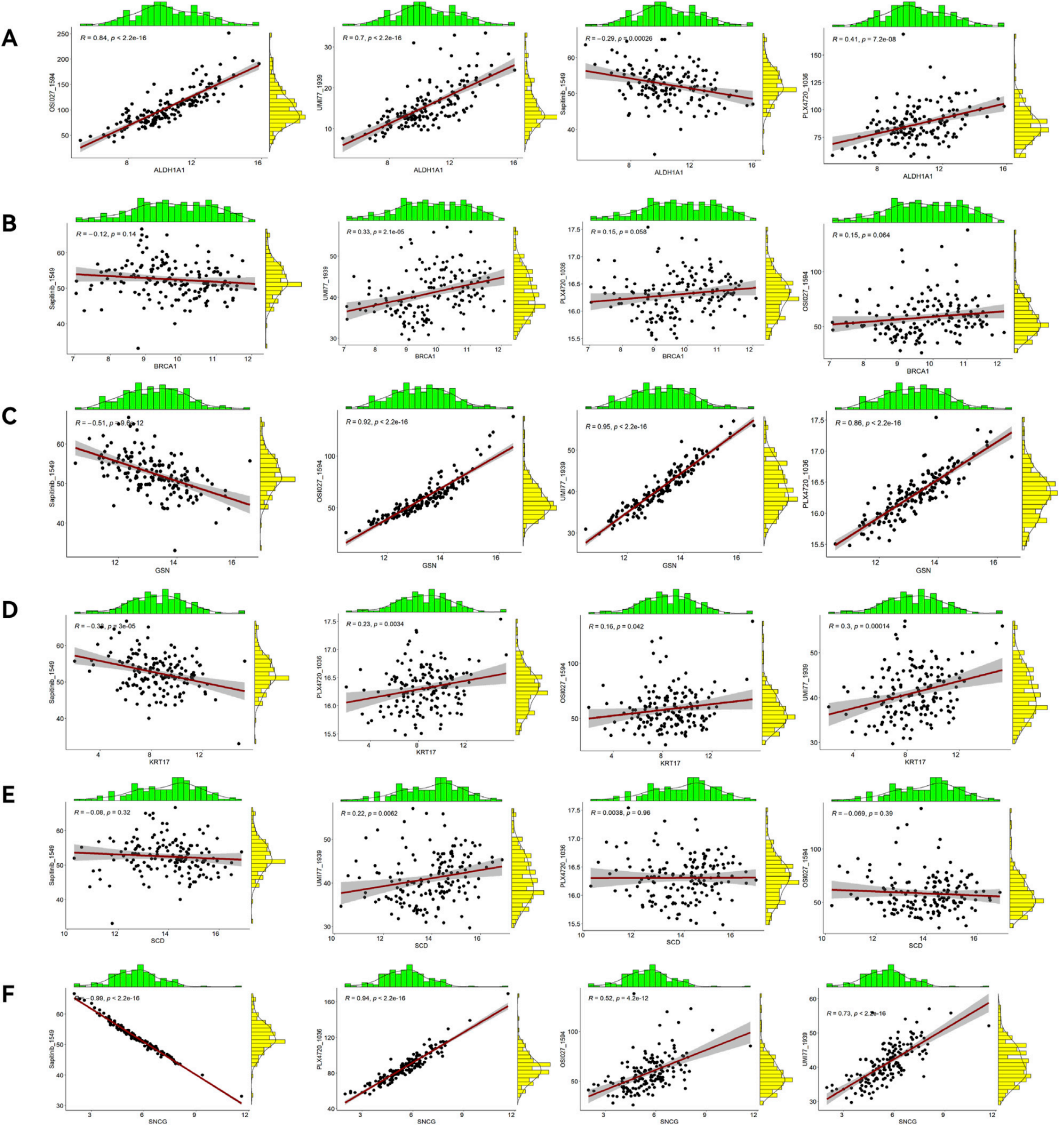
Correlation analyses between prognostic ARGs and drugs. Correlation analysis of ALDH1A1 **(A)**, BRCA1 **(B)**, GSN **(C)**, KRT17 **(D)**, SCD **(E)**, and SNCG **(F)**.

A sensitivity analysis for 198 drugs (Figures 12A–D) showed different IC50 values of four antitumor drugs OSI-027_1594, PLX-4720_1036, UMI-77_1939, and Sapitinib_1549 between the high-risk and low-risk groups. A correlation analysis (Figures 13A–F) showed that ALDH1A1, GSN, KRT17, and SNCG were positively correlated with the drugs OSI-027_1594, PLX-4720_1036, and UMI-77_1939, but negatively correlated with Sapitinib_1549. BRCA1 and SCD were positively correlated with the drug UMI-77_1939 and were not correlated with OSI-027_1594, PLX-4720_1036, or Sapitinib_1549.

### 3.8 Validation of prognostic genes

**FIGURE 14:**
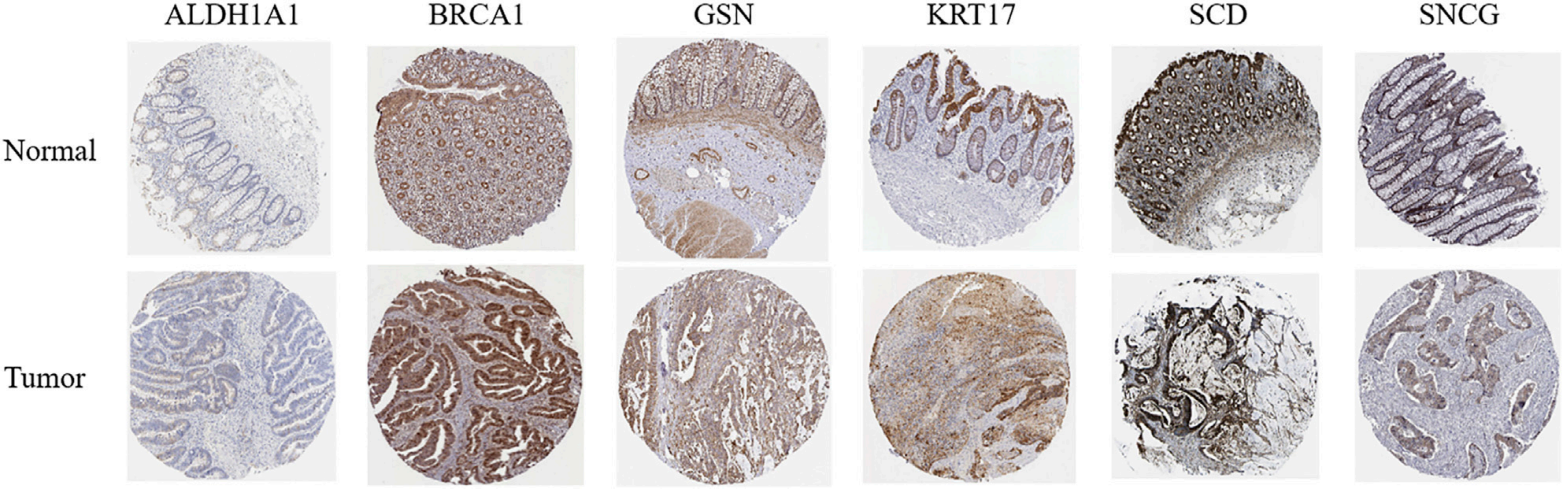
Protein expression levels of key ARGs in normal tissue and READ tissue, with data derived from the HPA online database.

**FIGURE 15:**
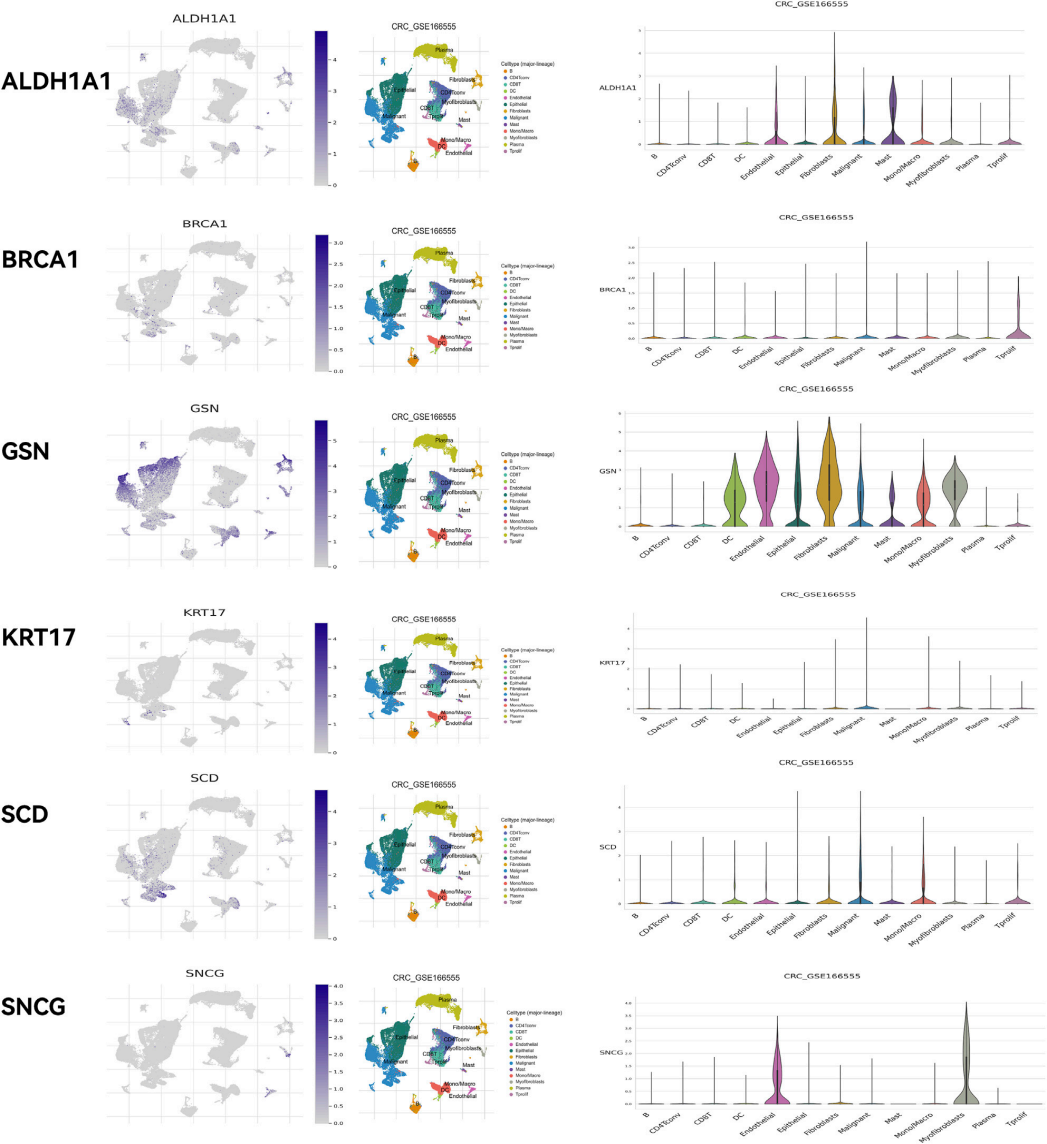
Validation using the TISCH2 online database.

HPA online database (Figure 14) demonstrated that ALDH1A1 was not detected in either normal rectal tissue or READ tissue but was moderately expressed in colon cancer tissue. BRCA1 was highly expressed in both normal rectal tissue and READ tissue. GSN was highly expressed in normal rectal tissue but was moderately or lowly expressed in READ tissue. KRT17 exhibited moderate expression in normal tissue and upregulation in READ tissue. SCD was highly expressed in normal tissue and either highly or moderately expressed in READ tissue. SNCG was highly expressed in normal tissue and moderately expressed in READ tissue. GEPIA online database (Supplementary Figure S4) showed that BRCA1, KRT17, and SCD were significantly upregulated in READ patients compared to normal individuals, while ALDH1A1, GSN, and SNCG were significantly downregulated in READ patients. In the GSE166555 dataset from the TISCH2 online database, cells were heterogeneous and interacted with each other in the TME (Figure 15). ALDH1A1 and GSN were primarily expressed in endothelial, epithelial, fibroblasts, and malignant cells; BRCA1 was primarily expressed in malignant cells, CD8T, and plasma cells; KRT17 was primarily expressed in fibroblasts and malignant cells; SCD was primarily expressed in epithelial, malignant, and mono/macro cells; and SNCG was primarily expressed in endothelial and myofibroblasts.

## 4 Discussion

Colorectal cancer is the fourth leading malignancy in the United States. Among adults under 50, colorectal cancer is the major cause of cancer-related death for men and the second major cause for women (Siegel et al., 2024). READ accounts for about 1/3 of all colorectal cancer cases, posing a significant threat to public health (Awiwi et al., 2023). In the past 20 years, READ treatment has been substantially improved, including novel chemotherapy, immunotherapy, and minimally invasive surgery (Emile et al., 2023). Nonetheless, the mortality rate is still high, suggesting the urgent need to find new targets and approaches. The pathogenesis of READ is complex and remains unclear. Anoikis, a specific mode of detachment-induced cell death associated with apoptosis, is an obstacles to cancer cell invasion (Raeisi et al., 2022). ARGs are associated with the development and prognosis of various cancers, including ovarian cancer (Qian et al., 2023), thyroid cancer (Tang et al., 2024), and renal cell carcinoma (He et al., 2023). To further explore the association between ARGs and the development and prognosis of READ, we constructed a prognostic risk model based on key ARGs in READ to investigate READ pathogenesis, assess its prognosis, and develop new therapeutic targets.

Differential analysis and Cox regression analysis identified six key ARGs (ALDH1A1, BRCA1, GSN, KRT17, SCD, and SNCG), and a prognostic risk model for READ was constructed based on these ARGs. Survival analysis and ROC curves validated the favorable predictive power of the model. External validation in the GSE87211 and GSE133057 datasets demonstrated that this model was effective in predicting the 5-year OS. The nomogram indicated that risk scores could accurately predict the 1-5-year OS rate of READ patients and can be used, along with age and TMN stage, as independent predictors of OS. The mechanisms of these key ARGs in tumorigenesis are complex, and some mechanisms have already been confirmed in cancer-related research. For example, Gorodetska et al. (2024) showed that ALDH1A1 regulated prostate cancer development and predicted metastasis and radioresistance in prostate cancer patients, potentially as a therapeutic target. Sale et al. (2020) used PCR to genotype breast cancer patients and found the involvement of BRCA1 mutations, suggesting that BRCA1 could serve as an early predictor of breast cancer onset. Patients with low GSN levels had greatly high 5-year relapse-free survival rates, and GSN expression was positively associated with colorectal cancer recurrence (Kim et al., 2018). KRT17 expression was significantly upregulated in urine RNA from bladder cancer patients (Dayati et al., 2024). Elevated SCD levels were detected in paclitaxel-resistant ovarian cancer patients, and pharmacological inhibition of SCD could suppress ovarian cancer cell proliferation (Ma et al., 2023). SNCG levels were notably elevated in the gastric juice and serum of gastric cancer patients, and SNCG was associated with tumor lymph node metastasis stages (Pan et al., 2022).

KEGG analysis showed that key ARGs were highly enriched in the PI3-Akt pathway, MAPK pathway, and microRNAs in cancer. These pathways are associated with tumorigenesis. The PI3-Akt pathway is highly active in cancer, and its activation is the core of the most unregulated metabolic pathways and satisfies the biosynthetic needs of rapidly growing cancer cells (Karim et al., 2022). Through whole-exome sequencing (Kumari et al., 2021), the PI3K-AKT pathway was identified as the main altered pathway in intestinal-type ampullary cancer. The MAPK pathway is crucial in ovarian cancer development, and MAPK inhibitors are applied for ovarian cancer treatment (Hendrikse et al., 2023). The MAPK pathway is frequently altered during disease progression and is significant in regulating drug sensitivity and tumor resistance (Lee et al., 2020). Abnormal expression of microRNAs in tumors either suppresses or promotes tumor growth (Mirzaei et al., 2021). Additionally, GO analysis showed that key ARGs were highly enriched in gland development, collagen-containing extracellular matrix, and secretory granule lumen, all of which may be related to anoikis.

GSVA analysis revealed that activated.CD4, activated.CD8, effector.memory.CD4, and Th17 were under-expressed in the high-risk group, while TGD and Treg were highly expressed. The ratio of activated CD4 T cells/Tregs <1 suggests poor prognoses in gastric cancer patients (Huo et al., 2024). Tumor cell ferroptosis, a new CD8^+^ T cell-mediated tumor clearance mechanism, is associated with immune-activated CD8^+^ T cells and lipid peroxidation via IFNγ, making tumor cells more sensitive to ferroptosis (Wang et al., 2019).

TNFRSF11B is associated with advanced lymph node metastasis and poorer survival outcomes in colon cancer patients, potentially by inhibiting memory-activated CD4^+^ T cell infiltration (Zhang et al., 2021). Salazar et al. (2020) stated that Th17 lymphocytes in the TME facilitated lung cancer metastasis. A high infiltration rate of TGD cells in tumor tissues is correlated with better clinical outcomes (Saura-Esteller et al., 2022). These studies suggest that the expression differences of these immune cells in the TME are important in tumor onset and prognosis. Further correlation analyses revealed considerable links between prognostic ARGs and immune cells, as well as strong correlations among different immune cells. Moreover, significant correlations were also observed between prognostic ARGs and immune checkpoint genes. Studies have also reported the correlation between these prognostic ARGs and immune checkpoint genes (Tu et al., 2019; Zhou et al., 2020; Castagnoli et al., 2019). These findings suggest that key ARGs in the model may influence tumor occurrence, development, and prognosis by modulating the immune TME.

Sensitivity analysis for 198 drugs found different IC50 values of OSI-027, PLX-4720, UMI-77, and Sapitinib between the high-risk and low-risk groups. Moreover, a notable correlation was revealed between prognostic genes and drugs. The antitumor effects of these four drugs have been confirmed in several studies. Mammalian target of rapamycin (mTOR) contributes to colon cancer progression, and OSI-027, as an mTOR inhibitor, suppresses tumorigenesis in colon cancer via the c-Myc/FOXO3a/PUMA axis (Lou et al., 2022). In HCC, PLX-4720 bound to AXIN1 to block tumor proliferation in TRAF2−/− mice on a high-fat diet (Li et al., 2024). UMI-77, as an effective Ku70/80 protein complex (Ku) inhibitor, disrupts bleomycin-induced DNA damage repair, sensitizes various cancer cells to DNA-damaging drugs, and considerably enhances the effects of other antitumor drugs (Chen et al., 2024). Additionally, the EGFR inhibitor Sapitinib can significantly strengthen the effectiveness of paclitaxel and doxorubicin in colon cancer and overcome resistance (Gao et al., 2020).

There are certain limitations. First, a prognostic risk model of key ARGs in READ was constructed, but we lacked experimental validation. More experiments may be needed to confirm the model and gene expression. Second, although READ patient samples were downloaded from the TCGA, our patient sample size was still insufficient. Third, there may be inherent biases in the data and algorithms, which could undermine the accuracy of the model.

## 5 Conclusion

In conclusion, a prognostic risk model is developed based on key ARGs in READ. Six prognostic genes are highly associated with READ and the TME. The model predicts READ prognosis, the TME and drug sensitivity. Therefore, these key ARGs could offer targets and reliable prognostic biomarkers for READ patients.

## Data Availability

The datasets presented in this study can be found in online repositories. The names of the repository/repositories and accession number(s) can be found in the article/Supplementary Material.
