# Supplementary figures and images for: Identification of anoikis-related genes to develop a risk model and predict the prognosis and tumor microenvironment in rectal adenocarcinoma
Source: Front Genet. 2025 Aug 18;16:1604541. doi: 10.3389/fgene.2025.1604541 (PMC12399626; doi:10.3389/fgene.2025.1604541)

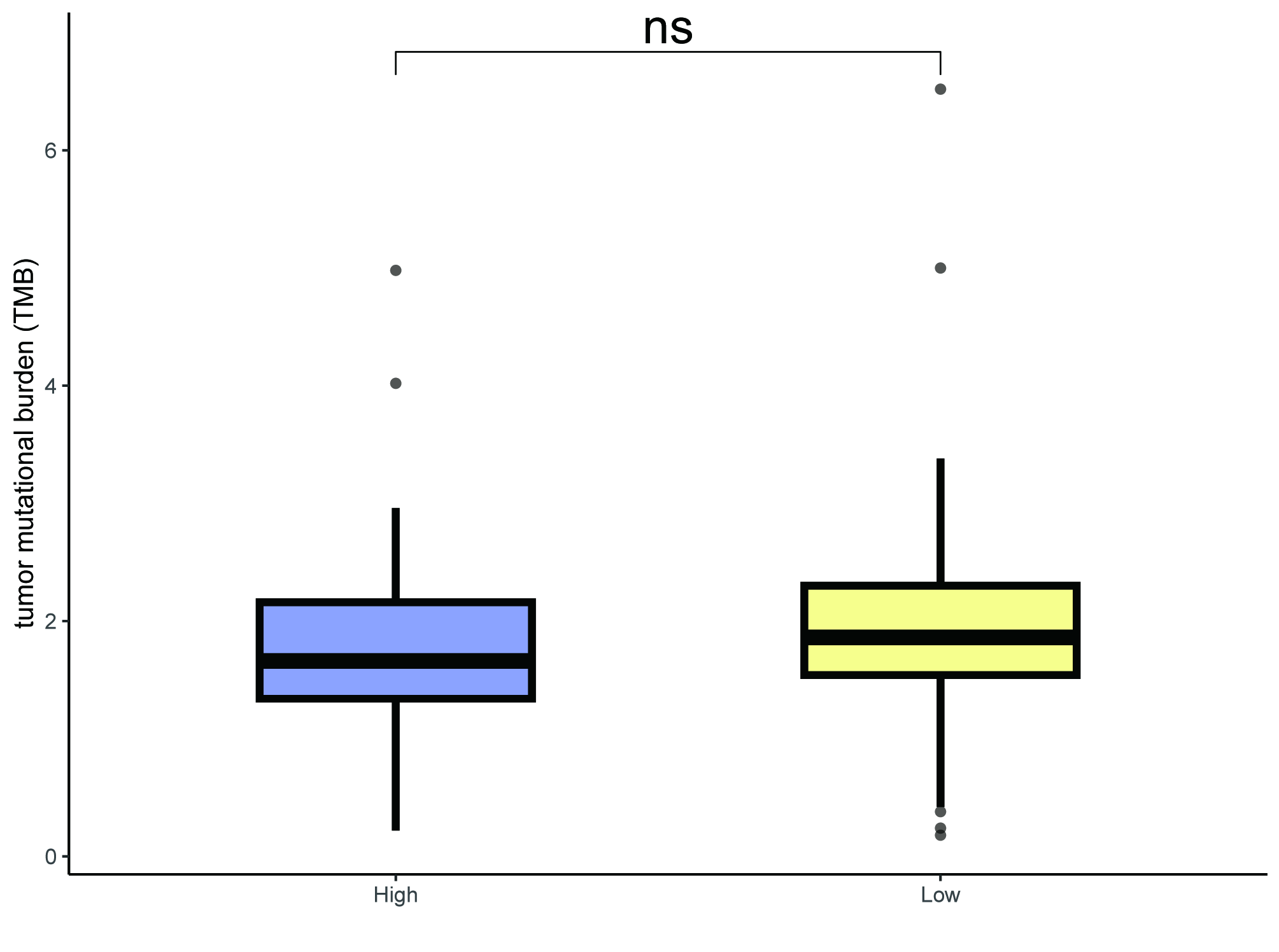

Supplement: Supplementary file 1 [file Image3.tif]

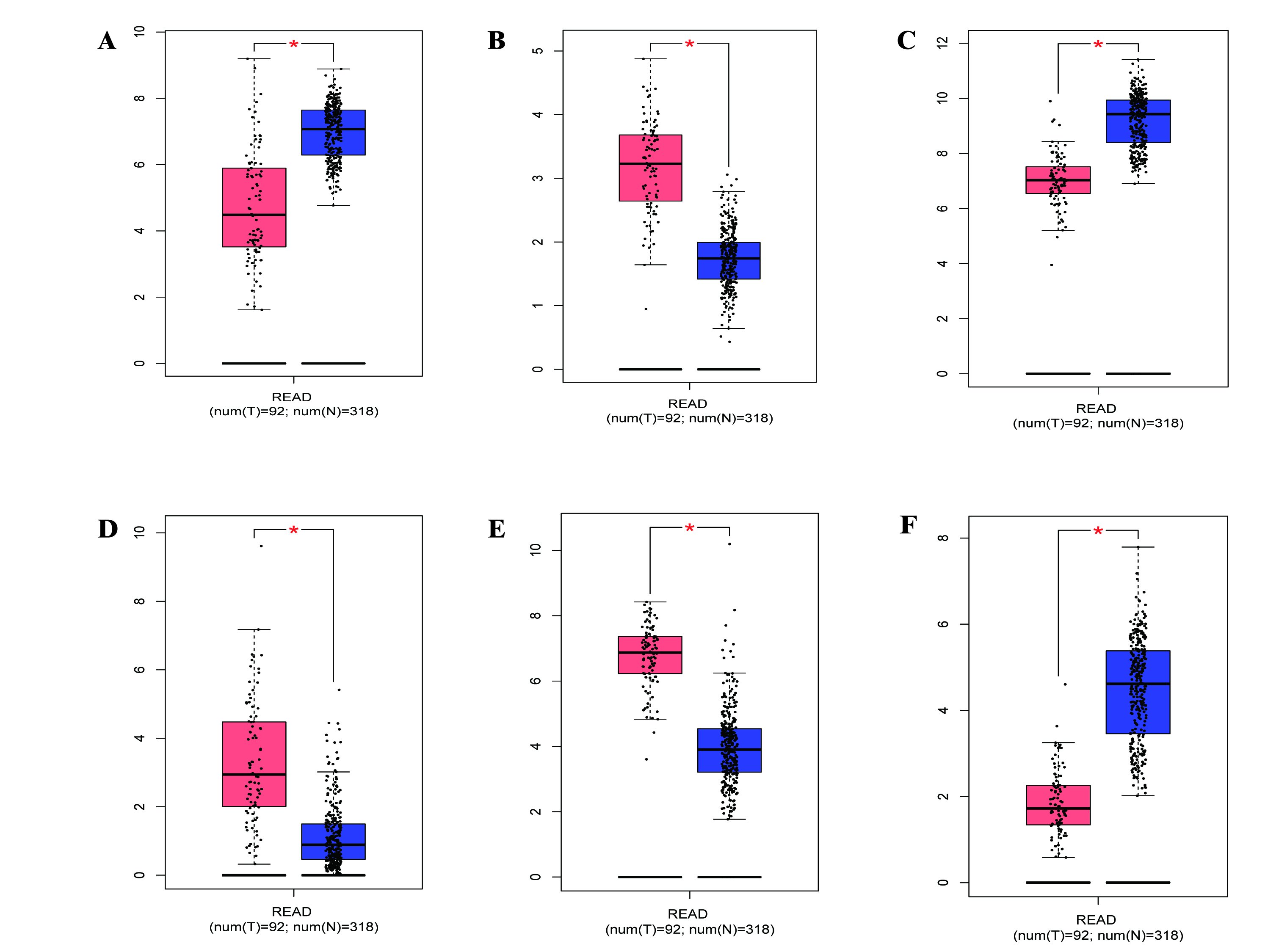

Supplement: Supplementary file 2 [file Image4.tif]

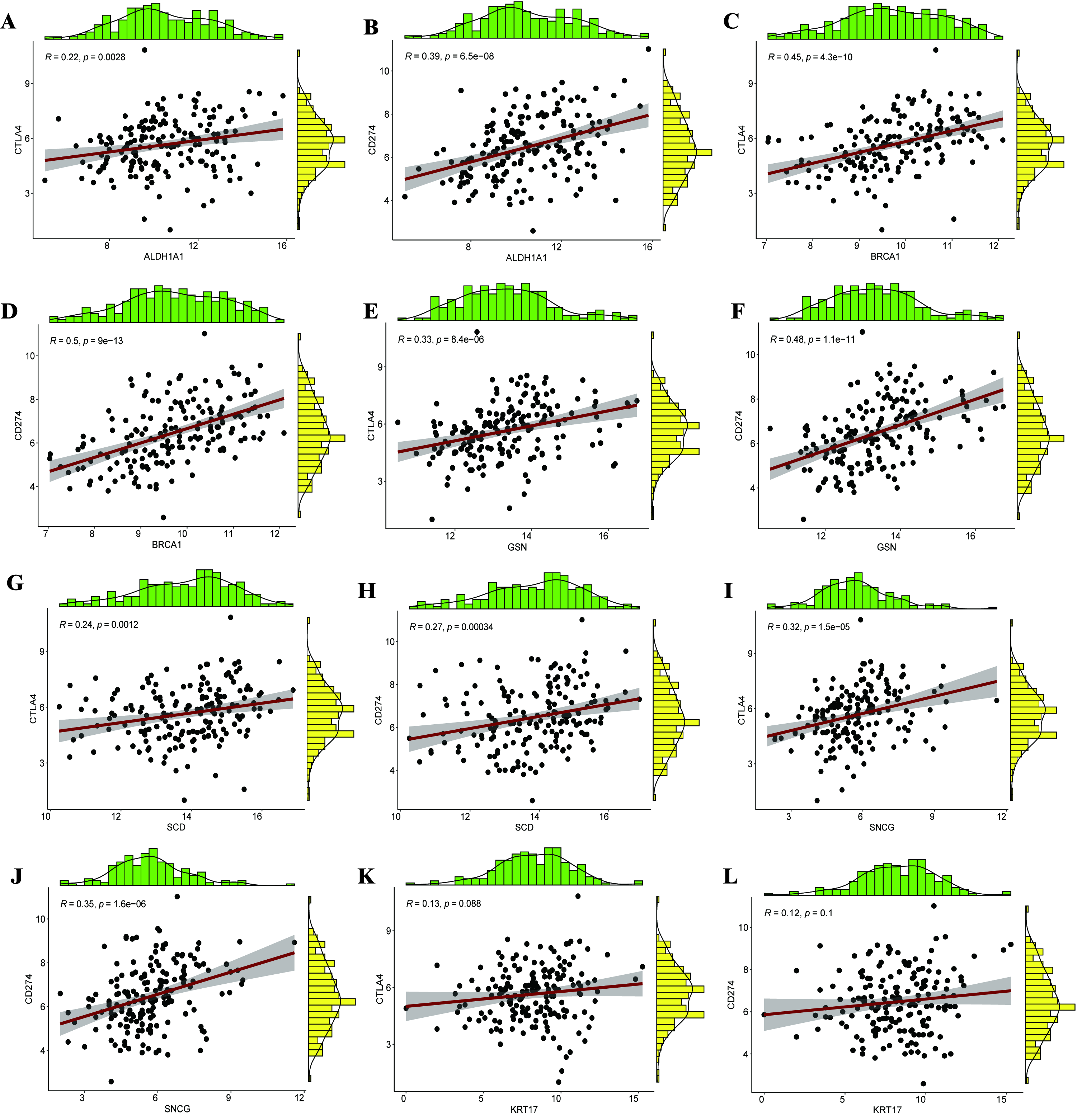

Supplement: Supplementary file 3 [file Image2.tif]

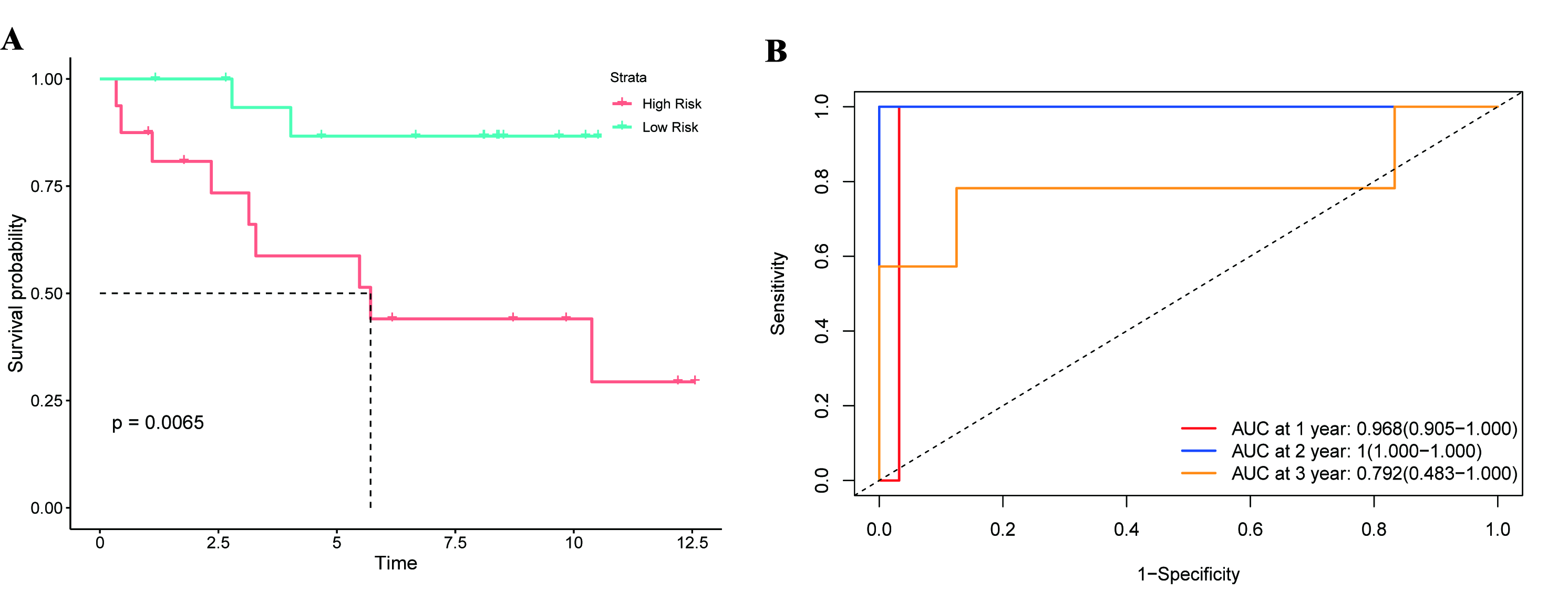

Supplement: Supplementary file 4 [file Image1.tif]
